# Recurrent intraductal papillary neoplasm of the bile duct due to intraductal dissemination: a case report and literature review

**DOI:** 10.1186/s40792-021-01318-0

**Published:** 2021-11-05

**Authors:** Yuki Nakayama, Takahiro Tomino, Mizuki Ninomiya, Ryosuke Minagawa, Yumi Oshiro, Takuma Izumi, Daisuke Taniguchi, Kosuke Hirose, Yuichiro Kajiwara, Kazuhito Minami, Takashi Nishizaki

**Affiliations:** 1 Department of Surgery, Matsuyama Red Cross Hospital, 1 Bunkyomachi, Matsuyama-shi, Ehime, 790-8144 Japan; 2 Department of Diagnostic Pathology, Matsuyama Red Cross Hospital, Ehime, Japan

**Keywords:** Intraductal papillary neoplasm of the bile duct, Biliary tumor, Recurrence

## Abstract

**Background:**

Intraductal papillary neoplasm of the bile duct (IPNB) is a subtype of biliary tumor. The 5-year survival rate of patients with IPNB who underwent curative resection is 81%. However, IPNB is known to often recur in other parts of the bile duct. Nevertheless, its mechanism remains poorly understood. Herein, we report the case of a patient with recurrent IPNB, which was considered to be attributed to intraductal dissemination in the common bile duct at 12 months after curative resection. We also made a review of the existing literature.

**Case presentation:**

A 69-year-old man was referred to our hospital for the evaluation and dilation of an intrahepatic bile duct (IHBD) mass. Computed tomography (CT) findings confirmed a mass in the left hepatic duct. Left trisectionectomy, extrahepatic bile duct resection with biliary reconstruction, and regional lymph node dissection were performed. Intraoperative examination of the resection margin at the common bile duct and posterior segmental branch of the hepatic duct was negative for the presence of malignant cells. Histologically, the tumor showed intraductal papillary growth of the mucinous epithelium and was diagnosed as non-invasive IPNB. It had a papillary structure with atypical epithelial cells lined up along the neoplastic fibrovascular stalks. Immunohistochemically, this was as a gastric-type lesion. At 12 postoperative months, CT revealed a 1.5-cm mass in the lower remnant common bile duct. We performed subtotal stomach-preserving pancreaticoduodenectomy. The tumor exhibited papillary growth and was microscopically and immunohistochemically similar to the first tumor. At approximately 16 months after the patient’s second discharge, CT showed an abdominal mass at the superior mesenteric plexus, which was diagnosed as recurrent IPNB. Chemotherapy is ongoing, and the patient is still alive. In this case, as described in many previous reports, IPNB recurred below the primary lesion in the bile duct.

**Conclusion:**

Based on our review of previous reports on IPNB recurrence, intraductal dissemination was considered one of the mechanisms underlying recurrence after multicentric development. Considering the high frequency and oncological conversion of recurrence in IPNB, regular follow-up examination is essential to achieve better prognosis in patients with recurrent IPNB.

## Background

Intraductal papillary neoplasm of the bile duct (IPNB) is classified as a biliary tumor subtype, according to the World Health Organization [1]. IPNB is an exophytic biliary epithelial tumor that historically includes various diseases, both benign and malignant [2]. It is considered as the biliary counterpart of intraductal papillary mucinous neoplasm of the pancreas (IPMN) [3]. According to the immunohistochemical profiles of mucin core proteins, IPNBs can be classified into four types: pancreaticobiliary, intestinal, gastric, and oncocytic types. The pancreaticobiliary type is the most common, whereas the oncocytic and gastric types are the rarest [4]. Lee et al. reported that patients with IPNB had good prognosis and that the 5-year survival rate of patients with IPNB who underwent curative resection was 81% [5]. However, IPNB often recurs in other parts of the bile duct. Rocha et al. [6] reported recurrence in 20 (51%) out of 39 patients with IPNB. Although several of these articles have reported that IPNB recurrence was multicentric, none provided pathological details regarding the recurrence pattern.

Herein, we report the case of a patient with recurrent IPNB because of intraductal dissemination in the common bile duct at 12 months after curative resection. We also review previous case reports on IPNB recurrence and discuss the mechanism of recurrence.

## Case presentation

**Fig. 1:**
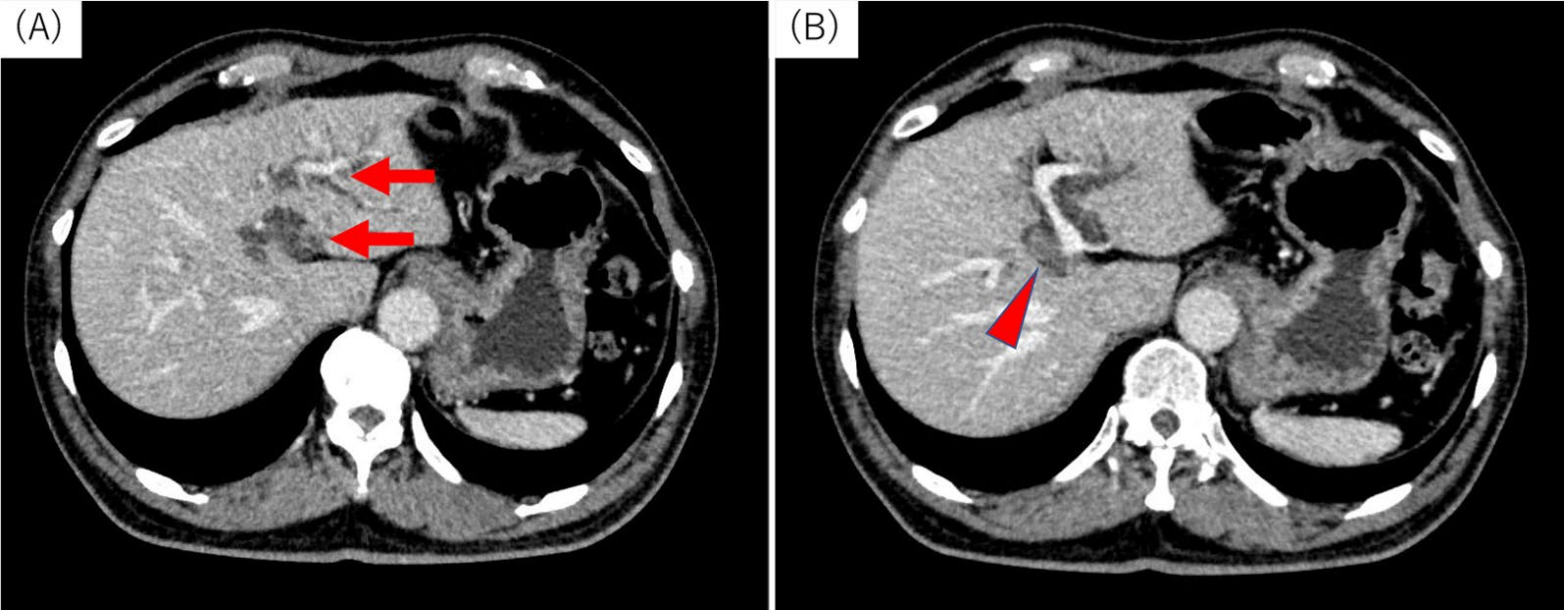
Contrast-enhanced computed tomography findings for the primary lesion. **A** Dilation of the intrahepatic bile duct was noted (arrow). **B** A mass of approximately 10 mm in diameter with sight enhancement was noted in the left hepatic duct (red arrowhead)

**Fig. 2:**
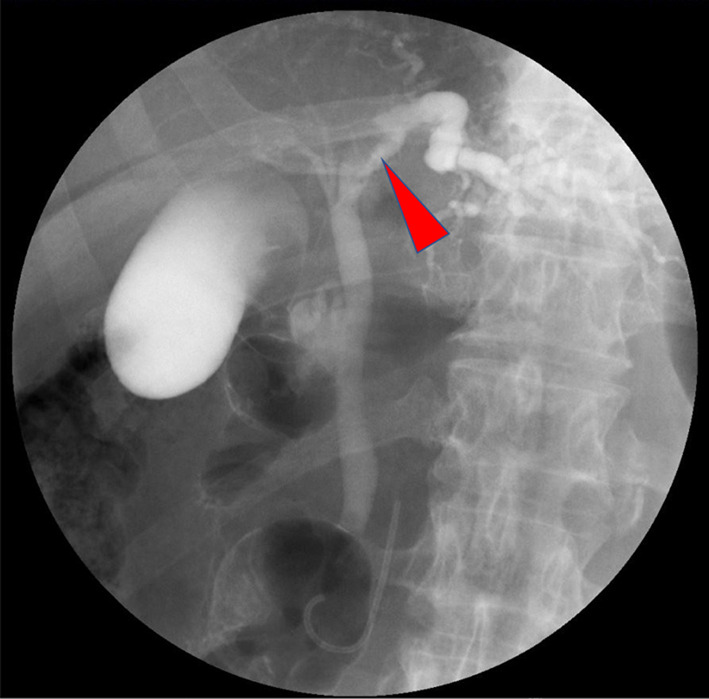
Endoscopic retrograde cholangiography. The arrowhead indicates a filling defect in the left hepatic duct. Simultaneously, bile duct brushing cytology was performed, but a clear diagnosis was not possible

A 69-year-old man was referred to our hospital for the evaluation and dilation of an intrahepatic bile duct (IHBD) mass. His initial laboratory values were as follows: total bilirubin, 1.0 mg/dL; direct bilirubin, 0.2 mg/dL; aspartate aminotransferase, 36 mg/dL; alanine aminotransferase, 50 mg/dL; alkaline phosphatase, 358 U/L; and γ-glutamyl transferase, 260 U/L. The tumor marker levels (including carcinoembryonic antigen and carbohydrate antigen 19–9) were within the normal ranges. Contrast-enhanced computed tomography (CT) revealed IHBD dilation in the left hemiliver (Fig. 1A) and a mass in the left hepatic duct (LHD) (Fig. 1B). The posterior segmental branch of the hepatic duct (PHD) draining into the LHD was also dilated. There was no evidence of distant metastasis. Magnetic resonance cholangiopancreatography (MRCP) revealed a 2-cm intraductal mass located between the LHD and PHD. Endoscopic retrograde cholangiography findings indicated the presence of a defect in the LHD and dilated LHD (Fig. 2), and bile duct brushing cytology was performed. Cytology showed atypical cells in the specimen from the mass, but the diagnosis was not clear.

**Fig. 3:**
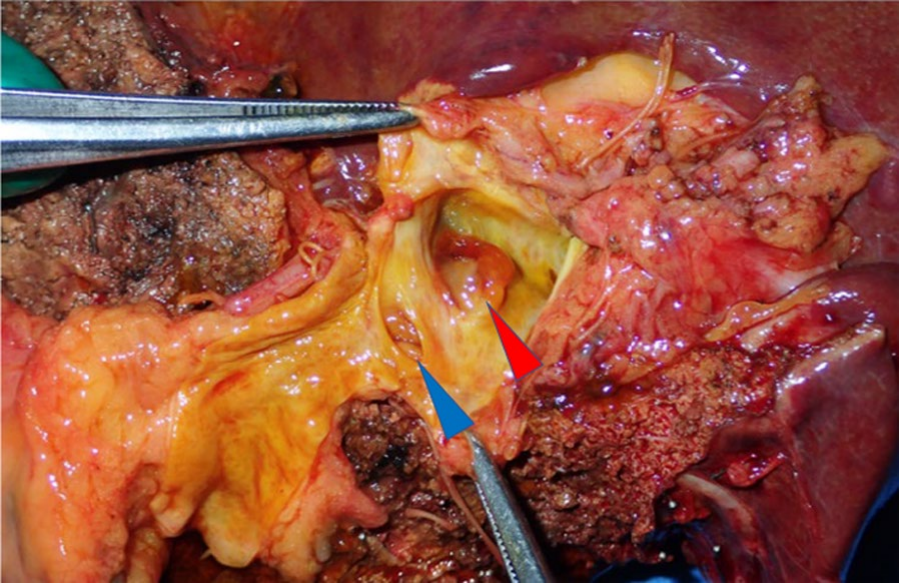
Photograph of the resected specimen (first surgery). The papillary tumor (red arrowhead) in the left hepatic duct was close to the opening of the posterior segmental branch of the hepatic duct (blue arrowhead)

**Fig. 4:**
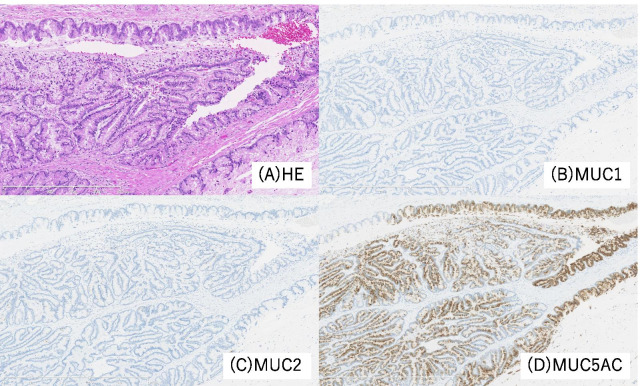
Histopathological findings of the primary lesion. **A** Hematoxylin and eosin staining findings. The tumor showed intraductal papillary growth of the atypical columnar epithelium with thin fibrovascular cores. The tumor cells contained abundant mucin and resembled crypt cells of the stomach. **B**–**D** Immunohistochemically, the tumor was MUC1- and MUC2-negative and MUC5AC-positive. These findings indicated that the tumor was of the gastric type

Brush cytology did not indicate that the tumor was an adenocarcinoma, but atypical cells were detected and the presence of adenocarcinoma could not be excluded. We performed left trisectionectomy, extrahepatic bile duct (EHBD) resection with biliary reconstruction, and regional lymph node dissection for curative resection. An elastic, hard papillary mass was found in the LHD. The patient had hepatic duct variation (PHD draining to the LHD), and the tumor was located close to the PHD. Concerning curative resection, excision of the PHD bifurcation was necessary. Intraoperative examination of the common bile duct and PHD resection margin was negative for the presence of malignant cells. By gross appearance, a papillary tumor was observed in the LHD (Fig. 3). Microscopically, the tumor showed intraductal papillary growth of the atypical columnar epithelium with thin fibrovascular cores. The cytological atypia was moderate to severe, but no invasion was detected. The patient was diagnosed as having IPNB. The atypical epithelium contained abundant mucin and resembled crypt cells of the stomach. Immunohistochemically, mucin 1 cell surface associated protein (MUC1) and MUC2 were negative, while MUC5AC and MUC6 were positive. Based on these findings, this lesion was regarded as of the gastric type (Fig. 4). There was no lymphovascular permeation or lymph node metastasis. The surgical margin in the permanent specimen was negative. The postoperative course was uneventful, and the patient was discharged at 21 days after surgery.

**Fig. 5:**
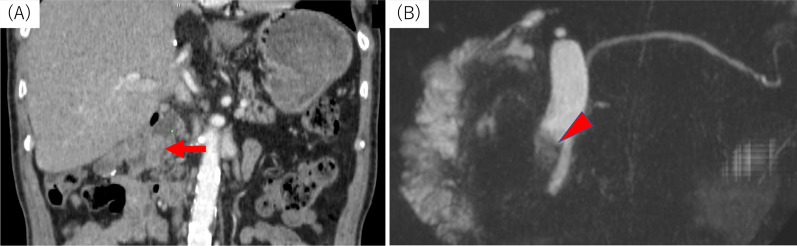
Contrast-enhanced computed tomography and magnetic resonance cholangiopancreatography findings for the recurrent lesion. **A** Contrast-enhanced computed tomography findings revealed a 1.5-cm mass in the lower remnant common bile duct at 16 months after the first surgery (arrow). **B** Magnetic resonance cholangiopancreatography findings identified the tumor as a defect in the lower remnant common bile duct (arrowhead)

**Fig. 6:**
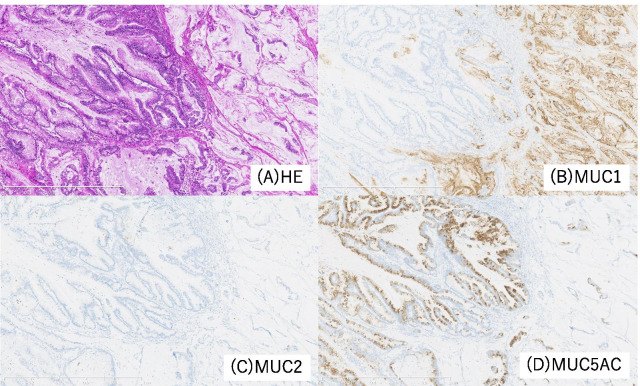
Histopathological findings of the recurrent lesion. **A** Hematoxylin and eosin staining findings. On the left side of the image, the recurrent tumor shows papillary proliferation that resembles the finding shown in Fig. 5A. Mucus production was observed in the atypical epithelial cells, as with the primary lesion. On the right side of the image, the tumor is expanding beyond the mucosa. **B** The tumor with a papillary structure was almost MUC1-negative; however, the tumor that exhibited invasion was MUC1-positive. **C**, **D** The tumor was MUC2-negative and MUC5AC-positive

After surgery, annual assessments involving blood tests and abdominal CT scans were planned. At 12 postoperative months, the patient visited our outpatient department with fever. Laboratory tests revealed elevated total bilirubin levels (1.6 mg/dL), but normal carcinoembryonic antigen (2.2 ng/mL) and CA19-9 (25.5 U/mL) levels. CT/MRCP showed a 1.5-cm mass in the lower remnant common bile duct (Fig. 5A and B). We suspected IPNB recurrence and planned surgical intervention. The patient underwent subtotal stomach-preserving pancreaticoduodenectomy. Intraoperative examination of the main pancreatic duct was negative for malignant cells. By gross appearance, the papillary tumor with rich mucus was apparent in the lower part of the common bile duct. Microscopically, the lesion resembled the lesion resected in the previous surgery. The tumor consisted of non-invasive and invasive portions, the former being similar to the primary lesion. Immunohistochemically, MUC1 and MUC2 were negative and MUC5AC and MUC6 were positive, indicating a gastric-type IPNB, as before. The latter part of the recurrent lesion showed conversion from the non-invasive to the invasive type, and was considered to have acquired MUC1-positive and MUC5AC-negative characteristics in the process (Fig. 6). Multiple metastases were detected in the posterior pancreatic lymph nodes. No nerve infiltration was observed, and the surgical margin in the permanent specimen was negative. A postoperative pancreatic fistula (International Study Group of Pancreatic Fistula grade B) was identified, but improved with antibiotic therapy. The patient was discharged at 28 days after surgery.

**Fig. 7:**
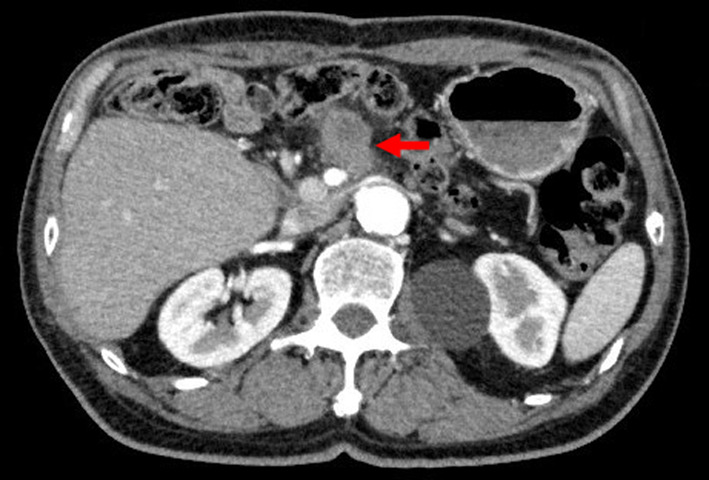
Recurrence at the superior mesenteric plexus. At 30 months after the first operation, contrast-enhanced computed tomography findings showed an abdominal mass at the superior mesenteric plexus (arrow)

At approximately 31 months after the first operation, CT findings revealed an abdominal mass at the superior mesenteric plexus (Fig. 7), which was diagnosed as recurrent IPNB. The patient received recurrent chemotherapy with gemcitabine, cisplatin, and S-1. However, at 6 months after the initiation of the first chemotherapy, he developed hematological toxicity and, consequently, he was switched to gemcitabine monotherapy. He is still alive at 42 months after the first operation.

## Discussion

We herein report the case of a patient with recurrent IPNB because of intraductal dissemination in the common bile duct at 12 months after curative resection. In this case, the primary and recurrent lesions were MUC1-/MUC2-negative and MUC5AC-/MUC6-positive, indicating gastric-type lesions. The standard treatment for IPNB is surgery, and previous studies have reported that a good prognosis can be obtained with R0 resection [7, 8]. However, the risk of recurrence is not low even after R0 resection [8, 9]. According to a report by Uemura et al. [7], 12 of 69 patients (17.4%) who underwent R0 resection relapsed. Regarding the mechanism of recurrence, IPNB has been reported to exhibit multicentric development [10]. In fact, metachronous IPMN recurrence in the pancreatic duct is known, and Izawa et al. reported multicentric IPMN recurrence with a genetical approach [11]. However, no reports have described multicentric IPNB recurrence with a genetical approach and, therefore, the mechanism of IPNB recurrence remains unclear. Yokode et al. [12] reported that the IPNB recurrence site is downstream of the initial lesion in most cases and dissemination is the main mechanism of IPNB recurrence concerning the flow of bile. They also reported that 84% of the first lesions were found in the IHBD, whereas 80% of the recurrent lesions were found in the EHBD. We searched PubMed using the terms “IPNB”, “recurrence”, and “biliary papillomatosis” and collected reports on cases of recurrent IPNB. A similar search was also conducted using ICHUSHI (http://login.jamas.or.jp/), a tool for searching for the medical literature written in Japanese. Table 1 [13–27] summarizes the cases of recurrent IPNB; including the present case, a total of 18 cases of recurrent IPNB were collected. In all cases, the initial treatment was surgery. Of the 12 patients for whom the resection margin in the initial surgery was noted in the report, 11 (91.7%) underwent R0 resection, but all 12 experienced IPNB recurrence. Of the 18 cases, 14 (77.8%) had a recurrence pattern from the upper site of the bile duct to the lower site. Of these, six cases mentioned the pathological type of the primary and recurrent lesions, all of which were of the same type.

In our case, the initial and recurrent lesions were diagnosed as IPNB, both of which were of the relatively rare gastric type [4]. The fact that these gastric-type lesions recurred at a downstream site suggested that the mechanism of IPNB recurrence may be intraductal dissemination. If multicentric development was the main mechanism of recurrence, the site of recurrence would be random. In addition, the fact that the pathology of the primary and recurrent lesions was the same in all cases, for which the information on pathological type was available, can be attributed to dissemination along the flow of bile. Of the 18 cases presented in Table 1, eight had presence or absence of mucin production for the initial and recurrent tumors, all of which had upper-to-lower site recurrence. Moreover, the presence or absence of mucin production was consistent between the initial and recurrent tumors. Among IPNBs, the mucin-producing type was reported to be 80% and 20% in IHBD and EHBD lesions, respectively [28]. In these cases, mucin production was observed in seven (87.5%) out of eight cases of recurrent tumors in the EHBD. If IPNB has multicentric recurrence, a high rate of mucin-producing type IPNB in the EHBD, as shown in Table 1, would contradict the low rate of mucin-producing type IPNB in the EHBD, as mentioned in previous works. This contradiction indicates that intraductal dissemination along the flow of bile is one of the main mechanisms of IPNB recurrence.

**Table 1:** The present case and 17 reported surgical cases of recurrent IPNB [10, 12–27]

		Primary lesion			Recurrence lesion							
References	Year	Primary surgery	Resection margin	Pathological type	Second treatment	Pathological type	Location of Primary lesion	Location of Recurrence lesion	Recurrence below the primary tumor	Mucin production (Primary/Recurrence)	Conversion from non-invasive type to invasive type	Recurrence after the second surgery (months)
[13]	1996	Lt. hepatectomy	Not listed	Not listed	Not listed	Not listed	IHBD	CBD	Yes	Not listed	Not listed	Not lised
[14]	2005	Lt. hepatectomy	Not listed	Not listed	Anterior segmentectomy/ extrahepatic bile duct resection	Not listed	LHD	CBD	Yes	Yes/Yes	Not listed	Not lited
[15]	2006	Extrahepatic bile duct resection	Negative	Not listed	PD	Not listed	Upper CBD	Lower CBD	Yes	Not listed	Yes	12
[16]	2007	Cholecystectomy	Negative	Not listed	PD	Not listed	Cystic duct	Lower CBD	Yes	Yes/Yes	No	No recurrence
[17]	2008	PD	Not listed	Not listed	Rt. hepatectomy	Not listed	Lower CBD	RHD	No	Not listed	Not listed	24
[18]	2009	Lt. hepatectomy/cadate lobectomy	Negative	Not listed	Tumor enucleation	Not listed	B4	CBD	Yes	Yes/Yes	No	28
[19]	2010	Lt. hepatectomy/caudate lobectomy/gall bladder resection	Not listed	Not listed	PD	Not listed	LHD	CBD	Yes	Not listed	Not listed	No recurrence
[20]	2013	Lt. hepatectomy/caudate lobectomy/extrahepatic bile duct resection	Negative	Not listed	PD	Not listed	LHD	CBD	Yes	Not listed	No	No recurrence
[21]	2013	Cholecystectomy	Not listed	Gastric	Resected(details unknown)	Gastric	Gall bladder	Lower CBD	Yes	Not listed	No	Not listed
[22]	2014	Lt. hepatectomy	Negative	Not listed	extrahepatic bile duct resection	Not listed	B2	CBD	Yes	Yes/Yes	Yes	35
[23]	2015	Rt. hepatectomy/caudate lobectomy/extrahepatic bile duct resection	Negative	Intestinal	PD	Intestinal	RHD	CBD	Yes	Yes/Yes	No	No recurrence
[10]	2015	Lt. hepatectomy/caudate lobectomy/extrahepatic bile duct resection	Negative	Pancreaticobiliary	stent detention	Pancreaticobiliary	LHD	RHD	No	Not listed	Not listed	–
[12]	2016	Rt. Hepatectomy	Positive	Intestinal	PD	Intestinal	RHD	CBD	Yes	Yes/Yes	No	No recurrence
[24]	2018	Lt. hepatectomy/caudate lobectomy/extrahepatic bile duct resection	Negative	Not listed	stent detention	Not listed	LHD-CBD	B5	No	Not listed	Not listed	–
[25]	2019	Lt. hepatectomy	No listed	Not listed	Not listed	Not listed	LHD	RHD	No	Not listed	Not listed	Not listed
[26]	2019	Extrahepatic bile duct resection	Negative	Not listed	Not listed	Not listed	CBD	Lower CBD	Yes	Not listed	Not listed	Not listed
[27]	2020	Rt. hepatectomy/caudate lobectomy/extrahepatic bile duct resection	Negative	Pancreaticobiliary	PD	Pancreaticobiliary	RHD	CBD	Yes	No/No	Not listed	No recurrence
Our case	2020	Lt. trisectionectomy	Negative	Gastric	PD	Gastric	LHD	CBD	Yes	Yes/Yes	Yes	19

*IHBD* intrahepatic bile duct, *LHD* left hepatic duct, *RHD* right hepatic duct, *CBD* common bile duct, *PD* pancreaticoduodenectomy, *Lt.* Left, *Rt.* right, *B2* Bile duct to liver segment 2, *B4* Bile duct to liver segment 4, *B5* Bile duct to liver segment 5

IPNB includes various benign and malignant lesions. The malignancy conversion rate of IPNB is 41–83% [29]. In our case, the primary lesion was non-invasive but the recurrent lesion had converted to the invasive type. In nine of the 18 cases, in which invasive type (or not) was mentioned, three cases (33.3%) experienced conversion from non-invasive type to invasive type in primary recurrence. All of the conversion cases recurred after the second surgery. Of the six non-conversion cases, only one case (16.7%) recurred. It is possible that conversion from the non-invasive to invasive type is related to the high rate of the second IPNB recurrence. In fact, the invasive type was reported to have inferior recurrence free survival compared to the non-invasive type [28]. Thus, even if R0 resection is performed on a lesion without invasion findings, a recurrent lesion in the lower bile duct could be detected as an invasive tumor, leading to recurrence after the second surgery.

As aforementioned, the main treatment for IPNB is surgery, but postoperative follow-up examination is also important. R0 resection for IPNB has a lower recurrence rate than R1 resection [9], and a better prognosis [4, 6, 30]. However, there is a good chance of recurrence even if R0 resection is performed. Considering the high frequency and oncological conversion of recurrence in IPNB, regular follow-up examination is essential for the early detection of IPNB recurrence.

## Conclusion

Based on our review of previous reports on IPNB recurrence, intraductal dissemination is considered one of the mechanisms underlying recurrence after multicentric development. Considering the high frequency and oncological conversion of recurrence in IPNB, regular follow-up examination is essential for better prognosis in patients with recurrent IPNB. Further studies to track and evaluate recurrent IPNB cases are warranted to comprehensively understand the oncology of IPNB.

## Data Availability

The datasets used and/or analyzed during the current study are available from the corresponding author on reasonable request.

## Abbreviations

IPNB: Intraductal papillary neoplasm of the bile duct
CT: Computed tomography
LHD: Left hepatic duct
PHD: Posterior segmental branch of the hepatic duct
MRCP: Magnetic resonance cholangiopancreatography
